# Identification of monogenic variants in more than ten per cent of children without type 1 diabetes-related autoantibodies at diagnosis in the Finnish Pediatric Diabetes Register

**DOI:** 10.1007/s00125-022-05834-y

**Published:** 2022-11-23

**Authors:** Minna Harsunen, Jarno L. T. Kettunen, Taina Härkönen, Om Dwivedi, Mikko Lehtovirta, Paula Vähäsalo, Riitta Veijola, Jorma Ilonen, Päivi J. Miettinen, Mikael Knip, Tiinamaija Tuomi

**Affiliations:** 1 New Children’s Hospital, Pediatric Research Center, University of Helsinki and Helsinki University Hospital, Helsinki, Finland; 2 Folkhälsan Research Center, Biomedicum Helsinki, Helsinki, Finland; 3 Research Program for Clinical and Molecular Metabolism, University of Helsinki, Helsinki, Finland; 4 Abdominal Centre, Endocrinology, University of Helsinki and Helsinki University Hospital, Helsinki, Finland; 5 Finnish Institute for Molecular Medicine, University of Helsinki, Helsinki, Finland; 6 Department of Pediatrics, PEDEGO Research Unit, University of Oulu, Oulu, Finland; 7 Department of Children and Adolescents, Oulu University Hospital, Oulu, Finland; 8 Medical Research Center, Oulu University Hospital and University of Oulu, Oulu, Finland; 9 Immunogenetics Laboratory, Institute of Biomedicine, University of Turku, Turku, Finland; 10 Translational Stem Cell Biology and Metabolism Research Program, Faculty of Medicine, University of Helsinki, Helsinki, Finland; 11 Tampere Center for Child Health Research, Tampere University Hospital, Tampere, Finland; 12 Lund University Diabetes Centre, Department of Clinical Sciences, Lund University, Lund, Sweden

**Keywords:** Diabetes in childhood, Finnish Pediatric Diabetes Register, Islet cell autoantibodies, MODY, Monogenic forms of diabetes, Next generation sequencing gene panel, Type 1 diabetes-related autoantibodies

## Abstract

**Aims/hypothesis:**

Monogenic forms of diabetes (MODY, neonatal diabetes mellitus and syndromic forms) are rare, and affected individuals may be misclassified and treated suboptimally. The prevalence of type 1 diabetes is high in Finnish children but systematic screening for monogenic diabetes has not been conducted. We assessed the prevalence and clinical manifestations of monogenic diabetes in children initially registered with type 1 diabetes in the Finnish Pediatric Diabetes Register (FPDR) but who had no type 1 diabetes-related autoantibodies (AABs) or had only low-titre islet cell autoantibodies (ICAs) at diagnosis.

**Methods:**

The FPDR, covering approximately 90% of newly diagnosed diabetic individuals aged ≤15 years in Finland starting from 2002, includes data on diabetes-associated HLA genotypes and AAB data (ICA, and autoantibodies against insulin, GAD, islet antigen 2 and zinc transporter 8) at diagnosis. A next generation sequencing gene panel including 42 genes was used to identify monogenic diabetes. We interpreted the variants in *HNF1A* by using the gene-specific standardised criteria and reported pathogenic and likely pathogenic findings only. For other genes, we also reported variants of unknown significance if an individual’s phenotype suggested monogenic diabetes.

**Results:**

Out of 6482 participants, we sequenced DNA for 152 (2.3%) testing negative for all AABs and 49 (0.8%) positive only for low-titre ICAs (ICA_low_). A monogenic form of diabetes was revealed in 19 (12.5%) of the AAB-negative patients (14 [9.2%] had pathogenic or likely pathogenic variants) and two (4.1%) of the ICA_low_ group. None had ketoacidosis at diagnosis or carried HLA genotypes conferring high risk for type 1 diabetes. The affected genes were *GCK*, *HNF1A*, *HNF4A*, *HNF1B*, *INS*, *KCNJ11*, *RFX6*, *LMNA* and *WFS1*. A switch from insulin to oral medication was successful in four of five patients with variants in *HNF1A*, *HNF4A* or *KCNJ11.*

**Conclusions/interpretation:**

More than 10% of AAB-negative children with newly diagnosed diabetes had a genetic finding associated with monogenic diabetes. Because the genetic diagnosis can lead to major changes in treatment, we recommend referring all AAB-negative paediatric patients with diabetes for genetic testing. Low-titre ICAs in the absence of other AABs does not always indicate a diagnosis of type 1 diabetes.

**Graphical abstract:**

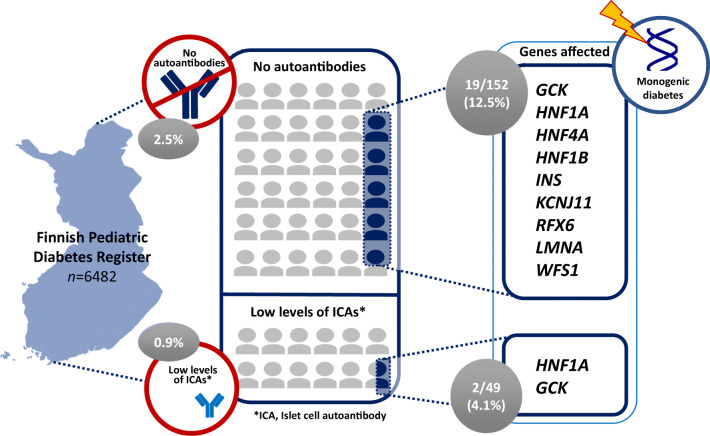

**Supplementary Information:**

The online version contains peer-reviewed but unedited supplementary material available at 10.1007/s00125-022-05834-y.

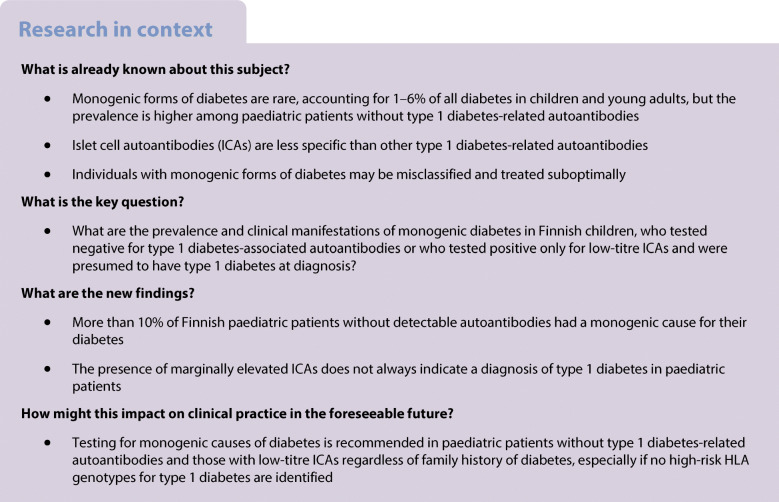



## Introduction

Large-scale genetic screening for monogenic diabetes in children and adolescents has not been performed. A prevalence of 2.1% was shown in an unselected childhood diabetes cohort (*n*=821) from Australia, while paediatric patients without type 1 diabetes-related autoantibodies (AABs) have a higher prevalence (4–15%) [1–3] especially in combination with residual C-peptide secretion (8–24%) [4, 5]. Monogenic diabetes includes very rare forms (neonatal diabetes [NDM], syndromic diabetes, monogenic autoimmune diabetes) but the most common form is MODY, which follows an autosomal dominant inheritance pattern. Individuals with MODY are often diagnosed with diabetes during late childhood, adolescence or early adulthood [6, 7]. While pathogenic variants in the genes encoding hepatocyte nuclear factor 1α (*HNF1A*) and glucokinase (*GCK*) are carried by more than 60% of the affected individuals [8], variants in more than ten genes have been conclusively associated with MODY [9].

A diagnosis of monogenic diabetes often enables personalised treatment. Some individuals may be switched from insulin to oral medication [10–12], especially those with pathogenic variants in *HNF1A*, *HNF4A* or genes encoding subunits of the ATP-sensitive potassium (K_ATP_) channel of the pancreatic beta cell (*KCNJ11* or *ABCC8*). The genetic diagnosis motivates familial testing and, in some cases, guides screening for comorbidities associated with the genetic finding.

Our main objective was to assess the prevalence and clinical manifestations of monogenic diabetes in individuals who were initially registered with type 1 diabetes in the Finnish Pediatric Diabetes Register (FPDR) but had no type 1 diabetes-related autoantibodies or had only low-titre islet cell autoantibodies (ICAs) at diagnosis. We also present two patients who underwent a successful switch from insulin to oral glucose-lowering agents years after the diagnosis of diabetes.

## Methods

### Study population

The FPDR covers more than 90% of children and adolescents aged ≤15 years diagnosed with diabetes in Finland since June 2002 [13]. In this study, we included 6482 participants of the FPDR initially registered with type 1 diabetes before November 2018 (mean±SD age at diagnosis 8.3±4.1 years, 43% female sex, estimated adult BMI [ISO-BMI, predicted by the BMI at registration, age and sex according to the Finnish growth centiles] 20.6±4.3 kg/m^2^). Most participants were ≤15 years at registration, but 23 had turned 16. For most patients, DNA samples and general information on the diagnosis of diabetes were available through the register (electronic supplementary material [ESM] Table 1). We studied the parents of those with gene variants putatively associated with diabetes if the parents had joined the register.

The sequencing and the mixed-meal tolerance test (MMTT) in an individual with a pathogenic variant in the *KCNJ11* gene were performed in collaboration with the FINNMODY study [14], which identified and characterised individuals with a suspected or established diagnosis of monogenic diabetes in Finland from 2014 onwards (www.botnia-study.org/finnmody). The results of the MMTT were compared with 45 control individuals without diabetes from the Botnia Study described previously [15]. The ethics committees of the Helsinki and Uusimaa Hospital District approved the studies which were conducted according to the Principles of the Declaration of Helsinki. All FPDR participants and/or their guardians signed an informed consent declaration. Individuals whose case reports are presented also agreed to their publication. Clinical details include minor modifications to maintain the participants’ anonymity.

### Laboratory analyses

#### Autoantibody status

Samples taken at diagnosis were tested for ICAs with indirect immunofluorescence or for autoantibodies against insulin, GAD, islet antigen 2 and zinc transporter 8 (IAA, GADA, IA-2A, ZnT8A) with specific radiobinding assays, as previously described [16]. The thresholds for positivity were as follows: ≥2.5 JDRF units (JDFU) for ICA; ≥1.57 relative units (RU) for IAA; ≥0.77 RU for IA-2A; ≥5.36 RU for GADA and ≥0.50 RU for ZnT8A.

#### HLA genotyping

The HLA class II genotyping was performed as previously described [17]. The HLA risk classification for type 1 diabetes based on the *HLA-DR/DQ* genotype frequencies observed in the Finnish population rests on disease risk associations of each haplotype, taking into consideration the synergistic effects of the *DR3-DQ2* and *DR4-DQ8* haplotypes. Each haplotype is classified as protective, neutral or susceptible. The highest risk was associated with the DRB1*04:01-DQA1*03-DQB1*03:02 haplotype, whereas the other common *DR4-DQ8* haplotype in Finland, DRB1*04:04-DQA1*03-DQB1*03:02, had a less strong disease association similar to the DRB1*03-DQA1*05-DQB1*02 haplotype [18].

#### MMTT

An MMTT composed of carbohydrates, fat and protein was consumed by participants within 10 min. Serial blood sampling was performed before and 15, 30, 60, 90, 120, 150 and 180 min after commencing the meal. See ESM Methods for details of the MMTT.

#### Next generation sequencing

In collaboration with the sequencing unit of the Institute for Molecular Medicine Finland, University of Helsinki, a next generation sequencing (NGS) gene panel was designed to include 42 genes reported to be associated with monogenic forms of diabetes, lipodystrophy or other glycaemic traits: *ABCC8*, *AKT2*, *APPL1*, *BLK*, *CEL*, *CISD2*, *DCAF17*, *DNAJC3*, *DYRK1B*, *EIF2AK3*, *FOXP3*, *GATA4*, *GATA6*, *GCK*, *GLIS3*, *HNF1A*, *HNF1B*, *HNF4A*, *IER3IP1*, *INS*, *INSR*, *KCNJ11*, *KLF11*, *LMNA*, *NEUROD1*, *NEUROG3*, *PAX4*, *PCBD1*, *PDX1*, *PIK3R1*, *PLIN1*, *POLD1*, *PPARG*, *PPP1R15B*, *PTF1A*, *RFX6*, *SLC19A2*, *SLC2A2*, *TRMT10A*, *WFS1*, *ZBTB20* and *ZFP57.* For *HNF4A*, the pancreatic transcript NM_175914.4 was used instead of the canonical transcript. The gene list from Ellard et al [19] was complemented from other sources such as the gene panel for monogenic diabetes from the leading laboratory in Exeter (https://www.diabetesgenes.org/, accessed 12 May 2022; previous versions of the panel assessed for the panel design). After excluding repetitive elements from the initial target region of 2,600,298 bp (the exons, flanking intronic regions [~±50 bp] and ~1 kbp region upstream of each gene), 19,084 probes were designed to cover a region of 2,285,454 bp. The samples, prepared with the Twist Enzymatic Fragmentation (EF) library (Twist Bioscience, South San Francisco, CA, USA), were run on the NovaSeq S2 platform with NovaSeq reagents (Illumina, San Diego, California, USA). The panel was successfully tested against known pathogenic or benign variants (*GCK* c.45+3A>G, c.544G>A, c.556C>T, c.563C>T, c.781G>A, c.823C>T, c.1198del, c.398_399insACATCTCTGAGTGCATCTCCGACT; *HNF1A* c.431T>C, c.779C>T, c.872dup, c.1501G>A, c.824_826del; *HNF1B* c.443C>T, c.721G>A, c.1474G>A; *HNF4A* c.225-8C>G, c.421C>T, c.691C>T, c.926G>A, c.256_257del; *RFX6* c.878_879del and *PDX1* c.226G>A, c.634G>A, c.716C>A) but it was not able to detect the *CEL* gene (encoding carboxyl ester lipase) variable number tandem repeat (VNTR) in the samples provided by A. Molven (University of Bergen). The mean read depth (the mean of the exonic mean depth per gene across samples) was 293, and for *HNF1A*, *HNF1B*, *HNF4A* and *GCK* it was 310, with 99.99% of the exonic targets covered by the read depth of 20 or more.

We confirmed the genetic findings reported in Table 1 in an accredited commercial laboratory (Blueprint Genetics, Espoo, Finland or HUSLAB Laboratory of Genetics, Helsinki, Finland).

**Table 1:** Monogenic variants identified in the study

Gene	OMIM identifier, inheritance model	Nucleotide change	Amino acid change	Age at diagnosis (years)	Parental history (diabetes / genotype)	ACMG classification
AAB-negative individuals						
*GCK*	125851, AD	NM_000162.5:c.364-2A>G		13	+ / not genotyped	P
*GCK*	125851, AD	NM_000162.5:c.863+1G>A		10	+ / +	LP
*GCK*	125851, AD	NM_000162.5:c.168del	p.(Met57CysfsTer30)	11	NA	LP
*GCK*	125851, AD	NM_000162.5:c.214G>A	p.(Gly72Arg)	8	NA	P (even without PP4 for the phenotype)
*HNF1A*	600496, AD	NM_000545.8:c.526+1G>A		13	+ / not genotyped	P (even without PP4 for the phenotype)
*HNF4A*	125850, AD	NM_175914.4:c.737T>A	p.(Ile246Asn)	15	NA	VUS (clearly suggestive phenotype)
*HNF4A*	125850, AD	NM_175914.4:c.112T>C	p.(Cys38Arg)	13	NA	VUS (clearly suggestive phenotype)
*HNF1B*	614527, AD	Chromosome 17q12 deletion		7	de novo	P
*HNF1B*	614527, AD	Chromosome 17q12 deletion		14	de novo	P
*KCNJ11*	618856 for NDM, AD; 616329 for MODY, AD	NM_000525.3:c.602G>A	p.(Arg201His)	2	de novo	P
*INS*	618858 for NDM, AD; 613370 for MODY, AD	NM_000207.2:c.94G>A	p.(Gly32Ser)	2	de novo	P
*INS*	618858 for NDM, AD; 613370 for MODY, AD	NM_000207.2:c.94G>A	p.(Gly32Ser)	2	de novo	P
*INS*	618858 for NDM, AD; 613370 for MODY, AD	NM_000207.2:c.163C>T	p.(Arg55Cys)	9	NA	LP (P if PP4 applied for the phenotype)
*INS*	618858 for NDM, AD, 613370 for MODY, AD	NM_000207.2:c.109G>A	p.(Glu37Lys)	14	de novo	VUS
*RFX6*	AD with reduced penetrance	NM_173560.4:c.878_879del	p.(His293LeufsTer7)	16	NA	LP
*WFS1*^a^	222300, AR	NM_006005.3:c.317T>A	p.(Val106Glu)	9		VUS^a^ (PP4 applied for the phenotype, see discussion)
*WFS1*^a^	222300, AR	NM_006005.3:c.862-1G>A				LP^a^
*WFS1*	222300, AR	NM_006005.3:c.1999C>T - hoz	p.(Gln667Ter)	9		LP
*WFS1*	222300, AR	NM_006005.3:c.1999C>T - hoz	p.(Gln667Ter)	5		LP
*LMNA*	150330, AD	NM_170707.4:c.1391_1396del	p.(Met464_Gly465del)	13	de novo	VUS
Low-titre ICA individuals						
*HNF1A*	600496, AD	NM_000545.8:c.872dup	p.(Gly292ArgfsTer25)	11	+ / not genotyped	P
*GCK*	125851, AD	NM_000162.5:c.757G>T	p.(Val253Phe)	14	NA	P

Except for the individuals homozygous for the *WFS1* p.(Gln667Ter) variant, all individuals were heterozygous for the identified variants. For the ACMG classification and related criteria, please refer to [22, 23] and https://clinicalgenome.org/affiliation/50016/, accessed 6 November 2021. ESM Table 2 presents the rest of the evidence used to classify the variants such as allele frequencies (to apply the criterion PM2_supporting), in silico predictions (the PP3 criterion) and previous publications

^a^These two variants were identified in one individual. Only the variants in *trans* can cause an autosomal recessive disorder but we could not test both parents to determine whether the variants were in *cis* or *trans*. Because of the phenotypic features including optic atrophy and diabetes, however, the individual has been included in the list

+, Positive parental history for diabetes/genotype; AD, autosomal dominant; AR, autosomal recessive; LP, likely pathogenic; NA, not available; OMIM, Online Mendelian Inheritance in Man (see www.omim.org, accessed 31 March 2022); P, pathogenic; PP4, supporting level of evidence for the phenotype by the ACMG criteria

#### Variant interpretation

We interpreted the variants in *HNF1A* according to the gene-specific criteria (https://clinicalgenome.org/affiliation/50016/, accessed 6 November 2021), applying the American College of Medical Genetics and Genomics (ACMG) criteria [20], and reported only likely pathogenic or pathogenic variants. For all other genes, we applied the general ACMG criteria [21], with modifications adopted from the *HNF1A*-specific criteria that either downgrade the weight of evidence or upgrade it especially for criteria relating to co-segregation [PP1] and for phenotype [PP4]. However, to apply *HNF1A*-specific rules for other genes is potentially conservative, as the modifications are not counterbalanced by additional gene-specific evidence. Therefore, we also report the variants of unknown significance (VUS) in non-*HNF1A* genes if the following criteria were met: (1) the individual’s phenotype was suggestive of monogenic diabetes; and (2) the variants had a low allele frequency in the gnomAD population database and were either protein-truncating variants, splice site variants predicted with a high SpliceAI score to alter splicing or a non-conservative missense variant with a REVEL score >0.7.

The statistical and graphical analyses were performed using SPSS Statistics V25.0 (IBM, USA) and R V4.1.2 [22].

## Results

Of 6482 participants in the FPDR, 162 (2.5%) were negative for all tested AABs at diagnosis, and 57 (0.9%) had only low-titre ICA (2.5–10 JDFU), referred to as the ICA_low_ group. DNA samples were available for 152 (94%) individuals in the AAB-negative group (2.3% of all participants) and for 49 (86%) individuals in the ICA_low_ group (0.8% of all participants).

In total, we report a gene finding associated with monogenic diabetes in 19/152 AAB-negative individuals (12.5% of the screened AAB-negative individuals and 0.3% of all 6482 children). Of these 19 patients, 14 had a gene variant classified as pathogenic or likely pathogenic by the ACMG criteria (Table 1). Four of these variants resided in *GCK*, five in *HNF1A*, *HNF4A* or *HNF1B*, four in the insulin gene (*INS*) and one in *KCNJ11*. Four individuals with variants in the laminin A/C (*LMNA*, *n*=1) or wolframin ER transmembrane glycoprotein (*WFS1*, *n*=3) genes were diagnosed with syndromic forms of monogenic diabetes. One individual had a protein-truncating variant in the regulatory factor X6 gene (*RFX6*), recently shown to be associated with MODY with reduced penetrance [23]. Interestingly, only four of these 19 individuals had a known family history of diabetes. Two in the ICA_low_ group had pathogenic variants in *HNF1A* and *GCK*, resulting in a monogenic diabetes prevalence of 4.1% (2/49) (Table 1).

The 19 AAB-negative individuals diagnosed with monogenic diabetes had a median (range) age at diagnosis of 9.6 (2.1–15.7) years and ISO-BMI of 19.6 (13.3–35) kg/m^2^. None had presented with significant ketosis or ketoacidosis at the time of diagnosis or carried a high-risk HLA genotype associated with type 1 diabetes (*p*=1.4965×10^−7^, χ^2^ test, compared with the AAB-negative individuals without a monogenic finding, with 15% having a high-risk HLA genotype and 16% presenting with ketoacidosis at diagnosis). The two individuals in the ICA_low_ group carried neutral HLA genotypes and were diagnosed as teenagers with mild hyperglycaemia without ketosis. Overall, the protective and neutral HLA genotypes regarding type 1 diabetes risk were more frequent in individuals without AABs in the FPDR than in those with AABs (protective, 12% vs 3%; neutral, 23% vs 15%) and risk genotypes were less common (high risk, 14% vs 25%) (*p*=0.000356, χ^2^ test). In the ICA_low_ group, protective HLA genotypes were found only in 2% of individuals. Further clinical details are presented in ESM Table 1.

We contacted all 21 patients with monogenic findings and contacted their treating hospitals. Eight (38%) individuals (*GCK* [four], *WFS1* [two], *HNF1B* [one] and *LMNA* [one]) had been diagnosed with monogenic diabetes during their clinical follow-up, irrespective of our study. In others, our register-based revision of diagnosis led to treatment changes. A diagnosis of *GCK*-MODY resulted in discontinuation of metformin in a patient misclassified as having type 2 diabetes. Despite initially presenting with insulin deficiency, a switch from insulin treatment to oral medication had been successful in four of five patients with findings in *HNF1A*, *HNF4A* or *KCNJ11* genes by the time of publication. We describe the process for two of them below.

### Patient with a VUS in *HNF4A*: transfer from insulin pump to oral glucose-lowering agent

A 13-year-old girl without a family history of diabetes presented with fatigue, obesity and hyperglycaemia (fasting plasma glucose 16.8 mmol/l, HbA_1c_ 103 mmol/mol [11.6%], no ketosis or acidosis). Relatively low C-peptide (0.6 nmol/l) in relation to hyperglycaemia had led to a diagnosis of type 1 diabetes. The girl was negative for AABs but the HLA genotype suggested a moderately increased genetic risk for type 1 diabetes. Insulin treatment was initiated. Four years after diagnosis, the total daily insulin administered via insulin pump was fairly low (0.6–0.7 U/kg) considering her age and ISO-BMI (28 kg/m^2^) and her C-peptide was surprisingly good (0.47 nmol/l at glucose 12.1 mmol/l).

**Fig. 1:**
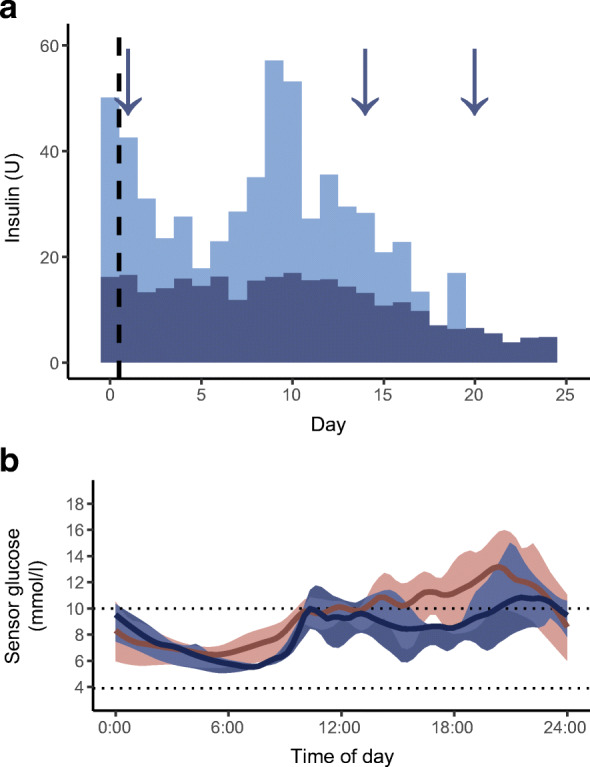
Patient with the *HNF4A* variant who was transferred from insulin pump to an oral glucose-lowering agent. (**a**) Bar plot showing the total daily dose of basal (dark blue bars) and bolus insulin (light blue bars) administered by the insulin pump, as well as the sequential initiation and dose increase of repaglinide at the major three meals of the day (indicated by arrows). Within 25 days, the patient gradually switched from insulin treatment to repaglinide. The vertical dashed line represents the initiation of the treatment transfer. (**b**) The 14 day mean level of sensor glucose measured by continuous glucose monitoring (Dexcom G6) during the treatment with the insulin pump (red line) and after the switch to repaglinide (blue line), with IQRs illustrated by the shaded area. Not only could the individual discontinue the insulin treatment but she also experienced fewer episodes of mild hypoglycaemia and improved her glycaemic control. The horizontal dotted lines represent the target range of glycaemia (3.9–10 mmol/l)

The sequencing revealed a heterozygous de novo variant in *HNF4A*. There was no information from the neonatal phase. Although the variant was classified as VUS, the bioinformatics (see Table 1) and clinical clues (no AABs, persisting C-peptide, low total daily insulin requirement) supported an empiric trial with repaglinide, a meglitinide drug with the same mechanism of action as sulfonylureas. Within 25 days, the girl was gradually switched from insulin to repaglinide (Fig. 1). Because the girl was overweight with suspected accompanying insulin resistance, metformin was initiated to further improve glycaemic control. After these treatment changes, she experienced fewer episodes of hypoglycaemia and displayed improved glycaemic control (decrease in HbA_1c_ from 74 to 53 mmol/mol [8.9 to 7.0%]).

### Transfer from insulin to sulfonylurea in a patient with *KCNJ11* p.(Arg201His)

A 2-year-old boy, born small for gestational age, with no hypoglycaemia in the neonatal phase, was admitted to hospital for hyperglycaemia during infection (no ketosis, HbA_1c_ 101 mmol/mol [11.4%], C-peptide 0.07 nmol/l). No AABs were detected, the HLA genotype risk group was neutral and he had no family history of diabetes. Insulin therapy was initiated. Within 2 years following diagnosis, he developed an absolute insulin deficiency with undetectable C-peptide. Fourteen years after the diagnosis, he was on an insulin pump with an average insulin requirement of 1.2 U/kg/day.

The gene panel revealed a heterozygous activating variant in the *KCNJ11* gene encoding for the Kir6.2 subunit of the K_ATP_ channel (Table 1), preventing its closure. In most cases, this results in permanent or transient NDM. This variant p.(Arg201His) can also cause *KCNJ11*-MODY with variable onset and severity of diabetes [24, 25].

**Fig. 2:**
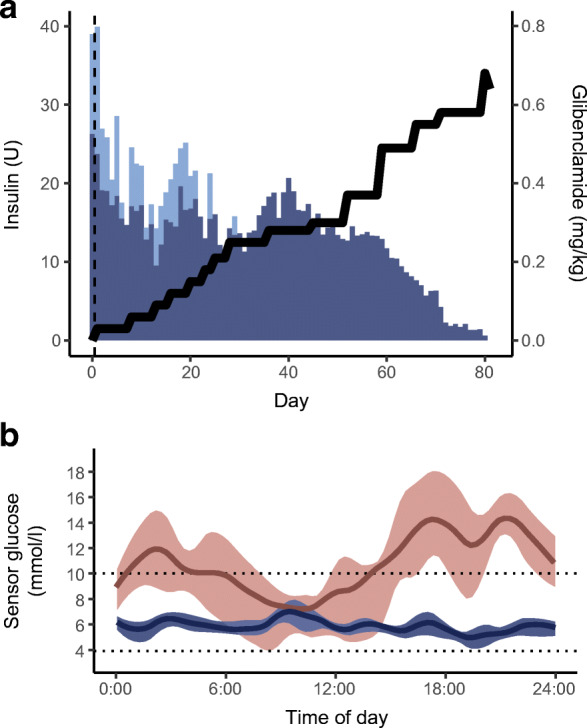
Patient with the *KCNJ11* variant who was transferred from insulin to a sulfonylurea. (**a**) Bar plot showing the total daily doses of basal (dark blue bars) and bolus insulin (light blue bars) and glibenclamide doses (black line, right-hand *y*-axis), along with increasing doses of glibenclamide. The insulin dose was gradually reduced and eventually the insulin treatment was discontinued after 80 days. The vertical dashed line represents the initiation of the treatment transfer. (**b**) The 14 day mean level of sensor glucose measured by continuous glucose monitoring (Dexcom G6) before the initiation of glibenclamide (red line) and after the switch to glibenclamide (blue line), with IQRs illustrated by the shaded area. The glycaemic control was remarkably improved by glibenclamide treatment. The horizontal dotted lines represent the target range of glycaemia (3.9–10 mmol/l)

As suggested by Pearson et al [26], we introduced treatment with an oral sulfonylurea (glibenclamide). The first dose of 0.03 mg/kg (1.75 mg), given 1 h before a standard MMTT, resulted in a hypoglycaemic event (requiring i.v. treatment with glucose) 30 min after the start of the meal. The insulin level increased dramatically after the first dose of sulfonylurea, indicating a rapid activation of endogenous insulin secretion from the beta cells. The C-peptide and proinsulin levels increased to a level similar to that seen in individuals without diabetes [15] (over 80 min, insulin increased from <1.4 to 1043.1 pmol/l, C-peptide increased from <0.003 to 2.07 nmol/l and proinsulin increased from <1.56 to 12.5 pmol/l) (Table 2). A minimal dose of 0.875 mg glibenclamide twice daily was first continued, then was doubled while the individual was an inpatient, and subsequently increased over several weeks up to 0.6 mg/kg per day. In parallel, the insulin dose was gradually reduced and discontinued 80 days later (Fig. 2).

**Table 2:** Glucose and hormone responses during repeated MMTTs performed for the boy with *KCNJ11*-MODY at the commencement of glibenclamide treatment and after discontinuation of insulin treatment and for control individuals without diabetes (*n*=45 as used in Dwivedi et al [15])

Time point	Individual with *KCNJ11*-MODY								Control individuals without diabetes				
	Glucose (mmol/l)	Insulin (pmol/l)	C-peptide (nmol/l)	Proinsulin (pmol/l)	Glucagon (ng/l)	GLP-1 total (pmol/l)	GLP-1 active (pmol/l)	GIP (pmol/l)	Glucose (mmol/l)	Insulin (pmol/l)	C-peptide (nmol/l)	Proinsulin (pmol/l)	GLP-1 total (pmol/l)^c^
First test													
M-60^a^	6.9	<1.4	<0.003	<1.56	8.6	8.5	<2.5	23.7	NA				
M0	8.2	319.1	0.662	3.1	14.1	11.1	2.63	9.1					
M20	5.8	1043.1	2.07	12.5	5.8	11.6	3.67	12.6					
M30	Sensor 3.8^b^												
Second test													
M-60													
M0	9.8	20.8	0.38	3.5	9.5	3.9	<2.5	5.8	5.4±0.5	57.6±31.4	0.66±0.21	5.5±3	25.6±7.3
M20	14	197.9	0.89	7.3	20.0	14.9	3.64		7.1±0.9	453.5±266.7	1.72±0.64		37±13
M40	14.5	363.2	1.56	15.3	15.6	13.6	5.37	42.4	6.6±1.6	572.3±259.1	2.35±0.66	27±10.2	36±11.6
M70	11.4	297.9	1.81	18.9	9.9	10.8	6.05	39.0	4.9±1.1	414.6±241.0	2.25±0.77		34.5±8.8
M100	8.7	366.7	2.35	25.5	10.7				4.9±1	324.3±196.5	2.01±0.67		35.7±9.2
M130	7.3	276.4	2.04	26.2	10.7	14.5	6.21	42.0	5.1±1	259.7±154.9	1.83±0.64	31.4±12.8	36±10.2
M160	7.6	217.4	1.8	23.5	11.7				5.2±1	171.5±120.2	1.55±0.64		32.9±10.9
M190	7.3	226.4	1.67	22.8	9.1	12.1	5.07	28.7	5.1±0.8	103.5±88.9	1.21±0.53		28.1±8.5

Data are shown for a boy with *KCNJ11*-MODY (age at MMTT 16 years, BMI 19 kg/m^2^) at the commencement of glibenclamide treatment and after discontinuation of insulin treatment. Data for control individuals without diabetes (49% female sex; mean±SD age 52.5±14.4 years and BMI 26±2.9 kg/m^2^) are presented as mean±SD

^a^60 min before the test meal, the individual with *KCNJ11*-MODY took 1.75 mg (first test) or 10.5 mg (second) of glibenclamide

^b^On the day of the first test meal, the basal insulin administered by an insulin pump was reduced to 1 U/h 60 min before the meal, then reduced to 0.5 U/h at 20 min and discontinued at 30 min when the patient experienced symptoms of hypoglycaemia and the test was stopped

^c^The method used for analysing total GLP-1 in the control individuals (RIA) differs from that used for the individual with *KCNJ11*-MODY (ELISA). For further details see ESM Methods

GIP, gastric inhibitory polypeptide; GLP-1, glucagon-like peptide-1; M, minutes before or after starting the test meal

On the sulfonylurea, the glycaemic control improved remarkably (time in range 3.9–10 mmol/l improved from 42% before the sulfonylurea to 98% with the sulfonylurea; CV for glucose changed from 46% to 17%).

## Discussion

Using a sequencing panel of 42 genes in the FPDR covering most Finnish paediatric patients diagnosed with type 1 diabetes since 2002, we identified a monogenic cause for diabetes in 12.5% of the AAB-negative individuals (9.2% with findings classified as pathogenic or likely pathogenic and 3.3% with VUS findings considered relevant). In addition, 4.1% of those positive only for low-titre ICAs had a monogenic cause.

The observed prevalence among individuals negative for five AABs was twice as high as the 6.5% reported in the Norwegian Childhood Diabetes Registry [2]*.* The difference is partly explained by only two AABs (GADA and IA-2A) measured in the Norwegian Registry but mainly by the included genes, as 8.5% of our AAB-negative individuals had pathogenic variants in *GCK*, *HNF1A*, *HNF4A*, *HNF1B* and *INS*, which were screened for in Norway. In addition, *INS* p.(Glu37Lys), a VUS reported in this study, is not a definitive finding (ESM Table 2). To interpret the relevance of any VUS in *INS* without conclusive prior functional variant-level evidence calls for caution as there is no unambiguous phenotype associated with heterozygous *INS* variants. However, as the variant turned out to be de novo, we included it on our list. If we focus on the most common MODY genes *GCK*, *HNF1A* and *HNF4A*, the prevalence in both our study (4%) and the Norwegian study (6%) is lower than the 15% seen in the Swedish National Cohort [3], likely explained by the latter including patients with all types of diabetes from paediatric clinics, with age at diagnosis of up to 18 years. In addition, a Lithuanian study including young adults up to 25 years of age showed a higher prevalence [27]. However, all these cohorts are demographically different, and the classification of variants has been variable. The inclusion of less symptomatic individuals would also increase the proportion of *GCK*-MODY that is associated with mild and life-long hyperglycaemia, as seen in the Lithuanian study.

Since the identification of ICAs [28], screening for type 1 diabetes-associated AABs has become routine in many countries. While the other AABs target specific proteins, the ICAs bind to various intracellular structures in the islets [29]. At the time of diagnosis, most insulin-deficient paediatric patients are positive for ICAs, although low ICA titres (≤10 JDFU) are also found in non-diabetic family members and in 4% of the Finnish general paediatric population [30, 31]. Hence, we extended the screening to the ICA_low_ individuals to reveal a monogenic cause for diabetes in 4.1% of this group. To date, it is not known whether pathogenic variants could potentially contribute to diabetes in some AAB-positive individuals.

Besides AAB negativity, clinical features in support of genetic testing have included low HbA_1c_, lack of type 1 diabetes-predisposing HLA genotypes, family history of diabetes and absence of ketoacidosis at diagnosis [32]. In support, and similar to the Swedish cohort, none of our studied individuals with monogenic diabetes had presented with severe ketosis or ketoacidosis. Further, most of the 19 individuals carried a protective or neutral HLA genotype regarding type 1 diabetes risk and none carried a high-risk genotype, whereas less than 20% in the whole FPDR carried a protective or neutral HLA genotype [33]. While a lack of risk genotypes should lead to considering alternative diagnosis to type 1 diabetes, we do not suggest using HLA typing to exclude patients from genetic testing for monogenic diabetes, considering the overall prevalence of risk HLA genotypes in the Finnish general population. On the other hand, 12 out of the 19 individuals would not have met the suggested HbA_1c_ criterion of <58 mmol/mol (7.5%) [3] (data not shown). We also noted that few of the individuals had reported a positive family history for diabetes and one-third had a confirmed de novo variant. All in all, only one individual scored high (75%) in the MODY probability calculator [34], whereas ten individuals with sufficient data for the calculation scored low (<20%). However, the prevalence of de novo genetic findings in our study is likely high, as those with a known family history of monogenic diabetes would presumably not have been registered as having type 1 diabetes in this register.

Variants in *GCK* and, surprisingly, *INS* were the most common causes for monogenic diabetes in our study, followed by variants in *HNF1A*, *HNF4A* and *HNF1B.* However, we are likely to underestimate the prevalence of *GCK*-MODY, as individuals with stable and mild hyperglycaemia might remain undiagnosed or they might be misclassified as having type 2 diabetes [35] or just not be registered in the FPDR. In the *HNF4A* gene, we report a novel missense variant, c.112T>C p.(Cys38Arg), graded as a VUS (Table 1). The successful transfer of the individual carrying this variant from insulin pump therapy to an oral short-acting meglitinide proves that she did not have type 1 diabetes but does not exclude the possibility of type 2 diabetes. However, the relatively low C-peptide level in relation to the degree of hyperglycaemia suggested mild insulin deficiency rather than resistance at diagnosis at the age of 13 years. We speculate that the loss of a cysteine (Cys) residue might destabilise the protein structure of hepatic nucleocyte factor 4α because the formation of a Cys-Cys disulfide bridge is hampered [36]. We also identified an individuals with an *RFX6* protein-truncating variant previously associated with MODY with reduced penetrance [23]. However, the variant p.(His293fs) might be far more prevalent in Finland than first assumed, as the FinnGen Study reported an allele frequency of 2.0×10^−3^ in Finland (https://r7.finngen.fi/variant/6-116916217-TAC-T, accessed 1 July 2022).

Identification of a monogenic cause for diabetes enables personalisation of the treatment. Dietary treatment involving balancing the carbohydrate intake can be sufficient in some forms of MODY. Individuals with *HNF1A*- and *HNF4A*-MODY or who carry *KCNJ11* gene variants [26, 37] can successfully be treated with a sulfonylurea [10, 38] or with sulfonylurea-like meglitinides [12]. However, after having witnessed a severe hypoglycaemic event in our patient with the *KCNJ11* variant upon the initiation of the sulfonylurea therapy, despite a lower first dose than suggested in published treatment protocols [26], we would advise starting sulfonylurea treatment under inpatient care. In our patient, the first dose of sulfonylurea triggered an immediate significant increase in proinsulin, C-peptide and insulin concentrations indicating their rapid release from beta cells. This was possibly enhanced by the suppression of glucagon secretion, suggested to be mediated though the paracrine effect of somatostatin, resulting in the loss of appropriate counter-regulation during insulin-induced hypoglycaemia [39]. The rapid insulin response with moderate C-peptide and proinsulin response preceding the severe hypoglycaemic event could indicate an uncontrolled release of previously produced insulin from a readily releasable pool of granules in beta cells, and not the newly synthesised hormone [40]. No hypoglycaemia occurred when the MMTT was repeated during sulfonylurea therapy, and the increase in proinsulin, insulin and C-peptide levels was tapered. The stimulation by incretin hormones was similar during both MMTTs, suggesting that these hormones have minimal impact in the development of hypoglycaemia.

In addition to allowing tailored treatment, a correct genetic diagnosis is crucial for further diagnostics and follow-up of possible comorbidities associated with syndromic forms of diabetes. *HNF1B* is associated with a multisystem disorder including renal manifestations, genital tract abnormalities, abnormal liver function, biliary cysts and neurological features [41]. *LMNA* is associated with familial partial lipodystrophy and, therefore, insulin resistance and diabetes as well as muscular diseases [42] (the individual with an *LMNA* variant in this study had a pre-established diagnosis of muscular disease). Wolfram syndrome (DIDMOAD) is a very rare disease involving diabetes mellitus, diabetes insipidus, blindness, deafness and progressive brainstem degeneration [43]. However, there is phenotypic variation in syndromic monogenic diseases, as also suggested by the individual with two *WFS1* variants and partial phenotype of Wolfram syndrome including diabetes mellitus and opticus atrophy (Table 1).

The study has some limitations. We screened only those paediatric patients included in the FPDR and registered initially with type 1 diabetes, who were negative for five different AABs or who had only marginally elevated ICAs. Although the FPDR reaches 90% of newly diagnosed children and adolescents with diabetes, 10% of the individuals with diabetes are lost and DNA samples were not available for all. Additionally, regardless of the register welcoming all kinds of diabetes, most research involving the FPDR concerns type 1 diabetes. Thus, paediatricians may be more likely to refer individuals with type 1 diabetes than those with a suspicion of other types of diabetes. Presumably, many individuals with known family history for monogenic diabetes are not registered in the FPDR and, in this study, we did not include samples from individuals who participated in a previously published study from Finland, in which participants were diagnosed with transient or permanent monogenic forms of diabetes before the age of 1 year between years 1980 and 2015 [44]. These facts, and the globally highest incidence of type 1 diabetes in Finland [45], explains to some extent the lower overall prevalence of monogenic diabetes found in our study (0.3%) compared with the published prevalence in other countries [3, 5, 46]. Specific repetitive targets such as *CEL* VNTR were beyond the performance of our gene panel. In addition, the panel did not include the genes for mitochondrial diabetes for technical reasons. Therefore, the true prevalence of monogenic forms among all paediatric patients diagnosed with diabetes in Finland might be somewhat higher than found here. In future, further investigation of the AAB-negative individuals will include whole exome sequencing with both nuclear and mitochondrial genes.

Early screening of monogenic diabetes in children with AAB-negative diabetes can have a major impact on the choice of treatment, enabling oral glucose-lowering treatment instead of insulin injections, with benefits on glycaemic control and long-term complications. Early targeted follow-up can be organised for individuals with syndromic forms of diabetes. Cost-effectiveness analyses, summarised recently by Naylor [47], have also shown that genetic testing for monogenic diabetes can be cost-effective or cost-saving in neonatal diabetes [48], in all paediatric patients presumed to have type 1 diabetes [49] and even young adult patients presumed to have type 2 diabetes [50].

In conclusion, our findings demonstrate the importance of determining the AAB status at diagnosis of diabetes in children and adolescents and justify testing for monogenic causes of diabetes in AAB-negative individuals and those with low-titre ICA regardless of family history of diabetes, especially if HLA genotypes conferring increased risk for type 1 diabetes are not detected.

## Supplementary Information

ESM (PDF 169 kb)

## Data Availability

The de-identified datasets generated during and/or analysed during the current study are available from the corresponding author on reasonable request.

## Abbreviations

AAB: Autoantibody
ACMG: American College of Medical Genetics and Genomics
FPDR: Finnish Pediatric Diabetes Register
GADA: GAD autoantibody
IA-2A: Islet antigen 2 autoantibody
IAA: Insulin autoantibody
ICA: Islet cell autoantibody
ISO-BMI: Estimated adult BMI in children
JDFU: JDRF units
K_ATP_: ATP-sensitive potassium
MMTT: Mixed-meal tolerance test
NDM: Neonatal diabetes
NGS: Next generation sequencing
RU: Relative unit
VNTR: Variable number tandem repeat
VUS: Variant of unknown significance
ZnT8A: Zinc transporter 8 autoantibody
