# Supplementary material for: Identification of monogenic variants in more than ten per cent of children without type 1 diabetes-related autoantibodies at diagnosis in the Finnish Pediatric Diabetes Register
Source: Diabetologia. 2022 Nov 23;66(3):438–49. doi: 10.1007/s00125-022-05834-y (PMC9892083; doi:10.1007/s00125-022-05834-y)
Supplement: Supplementary file 1 — (PDF 169 kb) [file 125_2022_5834_MOESM1_ESM.pdf]

ELECTRONIC SUPPLEMENTARY MATERIAL (ESM)

Identification of monogenic variants in more than ten per cent of children without type 1 diabetes-related autoantibodies at diagnosis in the Finnish Pediatric Diabetes Register

ESM Methods

Mixed meal tolerance test p. 2

ESM Table 1 p. 3

ESM Table 2 p. 4 - 7

ESM Methods

Mixed meal tolerance test (MMTT)

A mixed test meal (MMTT) composed of orange juice, coffee, slice of wholegrain bread, cheese spread, and marmalade was consumed within 10 minutes (1757 kJ [=420 kcal]; 69 g carbohydrate, 9 g fat, and 9 g protein). Serial blood sampling was performed before and 15, 30, 60, 90,120, 150, and 180 min after commencing the meal. During the MMTT, plasma glucose was analyzed using the Hemocue Glucose System (HemoCue, Ängelholm, Sweden), serum insulin and C-peptide using an electrochemiluminometric immunoassay on the Cobas e411 analyzer (Roche, Mannheim, Germany) and glucagon using ELISA (Mercodia, Uppsala, Sweden). Serum total and active plasma GLP-1 concentrations were determined using ELISA (Total GLP-1 NL-Elisa, Mercodia, Uppsala, Sweden, and Active form GLP-1, IBL, Kiehl, Germany) and serum GIP using ELISA (Millipore’s Human Total GIP, Merck, Darmstadt, Germany).

A radioimmunoassay method was used to analyse total GLP-1 in the control individuals without diabetes (Linco Research, Missouri, USA).

ESM Table 1. Clinical characteristics of the participants in the Finnish Pediatric Diabetes Register initially registered with type 1 diabetes (*n*=6482). The patients are grouped according to their autoantibody (AAB) status and gene panel findings.

	AAB+, All	AAB+: low-titre ICAs only	Low-titre ICAs with a gene finding	AAB- with a gene finding	AAB- without a gene finding
Total *n* (samples available for testing)	6320	57 (49)	2	19	133
Female, %	43	40	100	58	38
Age at diagnosis (years), mean (SD)	8.2 (4.0)	8.8 (4.2)	11; 14[a]	9.6 (2.1–15.7)[a]	10.6 (3.7)
BMI (kg/m^2)[b], mean (SD)	20.6 (4.3)	20.5 (3.6)	23.5; 19.3[a]	19.6 (13.3–35)[a]	21.2 (5.5)
Ketosis[c], %	38	28	0	0	29
Ketoacidosis[d], %	19	11	0	0	16
High-risk HLA, %	25	16	0	0	15
Neutral HLA, %	15	24	100	44	22
Protective HLA, %	3	2	0	39	9

[a] Data for patients with a monogenic finding are presented for each patient with positive low-titre ICA separately and in median (range) for the AAB-negative patients.

[b] Estimated adult BMI (ISO-BMI) predicted by the participants' actual BMI, age and sex according to the Finnish growth centiles.

[c] Blood 3-Hydroxybutyric acid > 3mmol/l

[d] pH < 7.3 and blood 3-Hydroxybutyric acid > 3mmol/l

ESM TABLE 2

Monogenic variants identified in the study. Table 1 and ESM Table 2 contain evidence for classifing the variants by the ACMG criteria.

Group	Gene	Nucleotide change	Allele frequecies (PM2_supporting applied?)	In silico predictions (PP3 applied?)	Other evidence and notes (including PMID in square brackets)
**AAB-negative**	*GCK*	NM_000162.5:c.364-2A>G	✓ (not present in gnomAD)	✓ ME: 5.7 -> -2.2, SpIAI: 0.99 (high precision) for acceptor loss	Acceptor loss -> PVS1_strong; [10447526], Bellanne-Chantelot, Saint-Martin unpublished (see [19790256]) -> PP1_moderate; *GCK*-MODY phenotype -> PP4_moderate
	GCK	NM_000162.5:c.863+1G>A	✓ (gnomAD 4.144×10^{6} / AC 1 in NFE with AF 9.224×10^{6})	✓ ME: 4.9 -> -3.3, SpIAI: 0.98 (high precision) for donor loss	Donor loss -> PVS1_strong; [19790256] reports c.863+1G>C with ME ALT -3.414 -> PM5_supporting; *GCK*-MODY phenotype -> PP4_moderate
	GCK	NM_000162.5:c.168del	✓ (not present in gnomAD)		Protein length change -> PM4; c.171del p.(Met57IlefsTer30) in [16965331] -> PS1_moderate; *GCK*-MODY phenotype -> PP4_moderate
	GCK	NM_000162.5:c.214G>A	✓ (gnomAD 3.978×10^{6}, AC 1 in AFR with AF 6.155×10^{5})	✓ S: 0.01 (deleterious), PP: 1 (probably damaging), REVEL: 0.994	Widely recognized variant around the world (for a few references, see [19790256]) -> PS4, PP1_Strong (functional data gives at least PS3_supporting)
	HNF1A	NM_000545.8:c.526+1G>A	✓ (not present in gnomAD)	✓ ME: 10.0 -> 1.890, SpIAI: 1.0 high precision for donor loss	Widely recognized variant around the world ([11272211], [17407387], [21242637], [18838325], [31754975], [18003757] etc.) -> PS4, PP1_Strong

HNF4A	NM_175914.4:c.737T>A	✓ (not present in gnomAD)	✓ S: 0 (deleterious), PP: 0.992 (probably damaging), REVEL: 0.950	The phenotype of neonatal hypoglycaemia and MODY type diabetes later at life clearly associated with *HNF4A* -> PP4_moderate; also, a conserved missense variant associated with a large Grantham distance (149); nearby codons affected in patients with MODY in [23348805]
HNF4A	NM_175914.4:c.112T>C	✓ (not present in gnomAD)	✓ S: 0 (deleterious), PP: 1 (probably damaging), REVEL: 0.981	See the patient case and discussion
HNF1B	Chromosome 17q12 deletion			Numerous reports including [16249435], [17924346] - definitely a pathogenic finding
HNF1B	Chromosome 17q12 deletion			Numerous reports including [16249435], [17924346] - definitely a pathogenic finding
KCNJ11	NM_000525.3:c.602G>A	✓ (not present in gnomAD)	✓ S: 0 (deleterious), PP: 1 (probably damaging), REVEL: 0.982	[12524280], [15583126], [15838686], [17446535] etc. -> PS4, functional data in these studies give at least PS3_supporting; a de novo variant with highly suggestive features -> PS3_moderate; patient's phenotype (see the patient case) -> PP4
INS	NM_000207.2:c.94G>A	✓ (not present in gnomAD)	✓ S: 0 (deleterious), PP: 1 (probably damaging), REVEL: 0.959	[17855560], [18162506], [19900242], [27634015], [26530398], [31605659] -> PS4_moderate, PP1_strong, [32994272] (an animal model) + [20034470], [19952343] (functional data) -> PS3_supporting, a de novo variant with clinical features -> PS2_supporting

INS	NM_000207.2:c.94G>A	✓ (not present in gnomAD)	✓ S: 0 (deleterious), PP: 1 (probably damaging), REVEL: 0.959	[17855560], [18162506], [19900242], [27634015], [26530398], [31605659] -> PS4_moderate, PP1_strong, [32994272] (an animal model) + [20034470], [19952343] (functional data) -> PS3_supporting, a de novo variant with clinical features -> PS2_supporting
INS	NM_000207.2:c.163C>T	✓ (gnomAD 4.041×10^{6} / AC 1 in NFE with AF 9.079×10^{6})	✓ S: 0 (deleterious), PP: 0.993 (probably damaging), REVEL: 0.824	[18192540], [20007936], [20226046], [20938745], [25721872] -> PS4_moderate, PP1_strong; functional data in [20007936] and [20724178] -> PS3_supporting
INS	NM_000207.2:c.109G>A	✓ (not present in gnomAD)	✓ S: 0.01 (deleterious), PP: 0.975 (probably damaging), REVEL: 0.725	Variants in nearby codons in patients with diabetes ([30915639], [18451997], [24411943], [25781672) -> PM1_supporting considered but not applied; A de novo variant with suggestive features -> PM3_supporting
RFX6	NM_173560.4:c.878_879del	for fully penetrant disorder - not met (gnomAD 1.842×10^{4} / AC 26 in FIN with AF 0.002449, AC 1 in NFE with AD 1.471×10^{5} and AC 1 in others with AF 4.803×10^{4})		[29026101]
WFS1	NM_006005.3:c.317T>A	✓ for AR disorder (gnomAD 4.241×10^{6}, AC 2 in FIN with AF 1.886×10^{4}, POPMAX filtering 0)	✓ S: 0 (deleterious), PP: 0.499 (possibly damaging), REVEL: 0.806 - PP3_supporting	Patients homozygous for a variant in the next codon 107 in [18806274] and [23103830]

		NM_006005.3:c.862-1G>A	✓ for AR disorder (gnomAD 3.987×10^{6}, AC 2 in NFE with AF 2.941×10^{5}, POPMAX filtering 4.880×10^{6})	✓ ME: 10.8 -> 2.0, SplAI 0.990 (high precision) for acceptor loss	Acceptor loss -> PVS1_strong; another patient homozygous for c.826G>A in [27124789]
	WFS1	NM_006005.3:c.1999C>T - hoz	✓ for AR disorder (gnomAD 5.981×10^{5} or 6.569×10^{6} in v.3.1.1)		[10521293], [17568405] -> PS4_moderate, PP1_moderate; functional data in [21454619] -> PS3_supporting; patient's phenotype -> PP4
	WFS1	NM_006005.3:c.1999C>T - hoz	✓ for AR disorder (gnomAD 5.981×10^{5} or 6.569×10^{6} in v.3.1.1)		[10521293], [17568405] -> PS4_moderate, PP1_moderate; functional data in [21454619] -> PS3_supporting; patient's phenotype -> PP4
	LMNA	NM_170707.4:c.1391_1396del	✓ (not present in gnomAD)		The genetic diagnosis was established before this study on the basis of the muscular disease associated with *LMNA*
**Low-titre ICA**	*HNF1A*	NM_000545.8:c.872dup	✓ (not present in gnomAD)		The most common variant in *HNF1A* to cause MODY, MDEP has reviewed the variant with PVS1, PP1_Strong, PS2_Moderate (ClinVar Variation ID 14927)
	GCK	NM_000162.5:c.757G>T	✓ (not present in gnomAD)	✓ S: 0 (deleterious), PP: 0.969 (probably damaging), REVEL: 0.948	[24735133], [26594346], Tuomi et al. unpublished data -> PS4_moderate, PP1_strong; *GCK*-MODY phenotype -> PP4_moderate; (A missense variant at a conserved position -> PP2 might be applied)

ACMG, American College of Medical Genetics; gnomAD, Genome Aggregation Database (followed by the allele frequency in gnomAD); AF, allele frequency; AC, allele count; NFE, Non-Finnish European; AFR, African/African America; ✓, (criteria) met; ME, MaxEntScan scores using the Maximum Entropy Principle; SplAI, SpliceAI score, S, SIFT score; PP, PolyPhen score; REVEL, Rare Exome Variant Ensemble Learner score; PMID, PubMed reference number; X -> Y, because of evidence X, Y was met; MDEP, Monogenic Diabetes Expert Panel, a ClinGen Variant Curation Expert Panel
